# Development of an artificial intelligence driven dose prediction pipeline for online adaptive magnetic resonance-guided radiotherapy

**DOI:** 10.1016/j.phro.2026.101048

**Published:** 2026-07-23

**Authors:** Benjamin Tengler, Moritz Schneider, Marcel Nachbar, Simon Boeke, Cihan Gani, Maximilian Niyazi, Paul Fischer, Christian F. Baumgartner, Daniela Thorwarth

**Affiliations:** a Section for Biomedical Physics. Department of Radiation Oncology, University Hospital and Medical Faculty, Eberhard Karls University Tübingen, Germany; b Department of Radiation Oncology, University Hospital Tübingen, Germany; c German Cancer Consortium (DKTK), partner site Tübingen, a partnership between DKFZ and University Hospital Tübingen, Germany; d Department of Biomedical Engineering, University of Basel, Switzerland; e Faculty of Health Sciences and Medicine, University of Lucerne, Switzerland; f Cluster of Excellence ‘Machine Learning in the Sciences’, University of Tübingen, Germany

## Abstract

**Background and Purpose:**

The closed-off nature of most treatment planning systems (TPS) limits the potential for using artificial intelligence (AI) tools during online adaptive treatments. The aim of this study was to develop an AI-driven pipeline (AutoAdapt) for online planning of adaptive radiotherapy usable in a closed-off setting, providing optimal plan constraints derived from a population-based dose prediction model.

**Material and methods:**

The AutoAdapt pipeline consists of a physics-aware Swin UNet transformer network for dose prediction trained on 266 magnetic resonance images from 25 prostate cancer patients treated with 60 Gy on a 1.5 T magnetic resonance linear accelerator. The predicted dose was used to calculate plan constraints that were subsequently fed into a commercial TPS. AutoAdapt was tested using ten unseen cases and compared to manual plans based on clinical objectives, time, and complexity.

**Results:**

While all plans were approved by a radiation oncologist, AutoAdapt met all clinical objectives in seven patients compared to ten when manually planned. AutoAdapt yielded a significantly lower D_0.035cm^3^_ to the rectum (*p* = 0.01). Manual plans achieved a median rectum V_20Gy_ of 44% compared to 51% in AutoAdapt plans (*p* = 0.02). The pipeline only required a median of 29 s (7.5%) longer than the manual planners.

**Conclusions:**

The developed pipeline resulted in high-quality plans, ready for clinical use without further adjustments. AutoAdapt prioritized maximum rectum dose over D_20%_ compared to manual planning, while requiring less manual work. In the future, AutoAdapt may be used to assist human planners and improve adaptive radiotherapy workflows.

## Highlights

• Pipeline for online adaptive MR-guided radiotherapy treatment planning.
• Comparison against manual planners for clinical criteria, time and complexity.
• All generated plans were clinically acceptable.
• Manual intervention reduced to approximately 20 s.
• Generated plans needed significantly fewer monitor units than manual plans.

## Graphical abstract

**Figure:**
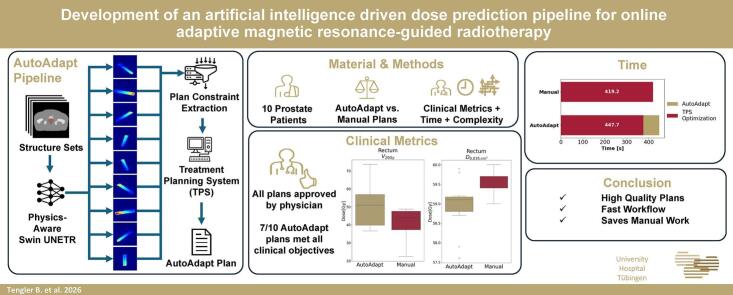

## 1 Introduction

Image-guided radiotherapy enables monitoring the changes in setup, anatomy, and tumor progression throughout the treatment cycle [1], [2]. However, this information is often solely used to adapt patient position by employing a couch shift [3]. In contrast, adaptation of radiotherapy plans based on information about anatomic changes or tumor progression was defined as adaptive radiotherapy (ART) [4]. The introduction of faster adaptation methods sparked the idea of online adaptive radiotherapy (oART), in which the treatment plan is adapted based on the observed changes while the patient lies on the treatment couch [5]. Different studies have already shown indications to reduce toxicity and improve local control by accounting for temporal changes in setup, anatomy, and tumor progression via oART [6], [7], [8], [9]. Depending on the imaging modality used, there may be additional benefits such as soft tissue contrast and live motion monitoring in magnetic resonance guided radiotherapy (MRgRT), while cone-beam computed tomography guided radiotherapy (CBCTgRT) potentially enables accurate electron density information and fast workflows [10], [11], [12].

While oART can provide many benefits, it also has its drawbacks in the form of longer treatment times and higher staff requirements, due to requiring a full treatment planning workflow, including imaging, segmentation, plan optimization, and quality assurance [13], [14], [15]. This requires the treatment planning process, which normally takes several hours, to be compressed into a few minutes. This is especially challenging for MRgRT, where the impact of the magnetic field on radiation transport must be considered, resulting in longer calculation times despite speed optimization based on graphical processing units (GPUs) and the use of intensity-modulated radiotherapy (IMRT) [16]. In a previous study, we found that large anatomical changes to the reference plan require adjustment of the plan constraints, while minor changes can be handled by the treatment planning system (TPS) without plan constraint adjustments [17].

For a fast manual plan adaptation, a good grasp of the underlying optimization principles and experience with the TPS are required, where some centers stated that up to nine months of prior treatment planning practice was necessary before colleagues were experienced enough for online adaptive planning [18].

By automating the online adaptive planning process or predicting patients where adaptation is needed, one could accelerate planning and reduce interaction time [19], [20]. This approach has already demonstrated some potential, utilizing knowledge-based planning and multicriteria optimization [21], [22], [23]. Pakharel et al. [24] have investigated a commercial solution for automated treatment planning based on a hierarchical optimization approach, achieving fast treatment options while, however, limiting further fine-tuning efforts by the user, especially if the adapted plan is deemed clinically unusable. In contrast, Naccarato et al. [25] implemented a lexicographic optimization guided by a wish list that could be integrated into the online workflow while still allowing further manual adaptation. Kalifa et al. [26], [27] went a step further by creating applicable treatment plans using dose mimicking, but these could not be used online, as the system did not support the use of externally generated treatment plans. Without access to the system, the only way to interact with the optimizer is via the plan constraints. Therefore, Liu et al. [28] investigated a pipeline that transforms dose prediction results into plan constraints that can be manually fed back into the TPS. While they achieved a similar performance as the manual planer, they had to rely on a patient-specific dose prediction model that needed to be retrained for each patient, as the population-based model was not accurate enough. Although this was helpful to increase model performance, it introduced the problem of how to commission a model that changes for every patient. Unlike Liu et al. [28] a physical component was introduced into the loss function of a population-based dose prediction model to improve its accuracy and render the patient-specific model unnecessary.

The aim of this study was to develop a pipeline that uses dose prediction to automate planning and can be integrated into the standard MRgRT workflow. The pipeline was tested by comparing its performance with manual planning in terms of clinical criteria, execution time, and plan complexity.

## 2 Materials and methods

### 2.1 Dose prediction model

The dataset for model creation consisted of 266 magnetic resonance image (MRI) datasets from adaptive clinical MRgRT fractions of 25 prostate cancer patients treated on a 1.5 T magnetic resonance linear accelerator (MR-Linac) (Unity, Elekta, Stockholm, Sweden) with a prescription dose of 20 × 3 Gy using 9-beam IMRT. Only reoptimized fractions based on the daily anatomy were included. All patient data was acquired as part of a phase II trial approved by the internal review board (NCT041722753), and patients gave written informed consent to the academic use of trial data. To generate high-quality training data, treatment plans were automatically created for each fraction using a particle swarm optimization approach as previously described in Tengler et al. [17]. The dataset was separated into a training dataset of 216 fractions from 17 patients and a validation set of 50 fractions from 8 unseen patients.

A Swin UNEt Transformer (Swin UNETR) architecture that uses transformer structures in the encoder was employed to improve global feature extraction (cf. Supplementary material A). The model was trained based on the training dataset, receiving a total of five input maps, consisting of one for planning target volumes normalized on the highest dose level (PTV60, PTV57.6), three organs-at-risk (OAR) (rectum, bladder, and urethra), and a bulk density assigned computed tomography (CT) [29].

To improve explainability and safety against out-of-distribution cases, the model was designed to output nine direction-specific dose distributions. By doing so, a gradient loss term was implemented to ensured that the model conformed to physical laws –in this case, the depth-dependent dose decay. The gradient loss was combined with a root mean square error (RMSE) for spatial features and optimization guidance, dose-volume histogram (DVH) difference for clinical features, and plan constraint difference for optimization features. The training was performed on an Nvidia A6000 with a cosine annealing learning rate of 0.001, stopping after 500 epochs without improvements in the validation set, running to a total of 1164 epochs. The model and technical specifics are further characterized using the AID-RT model card in the Supplementary Material [30].

### 2.2 AutoAdapt

**Fig. 1:**
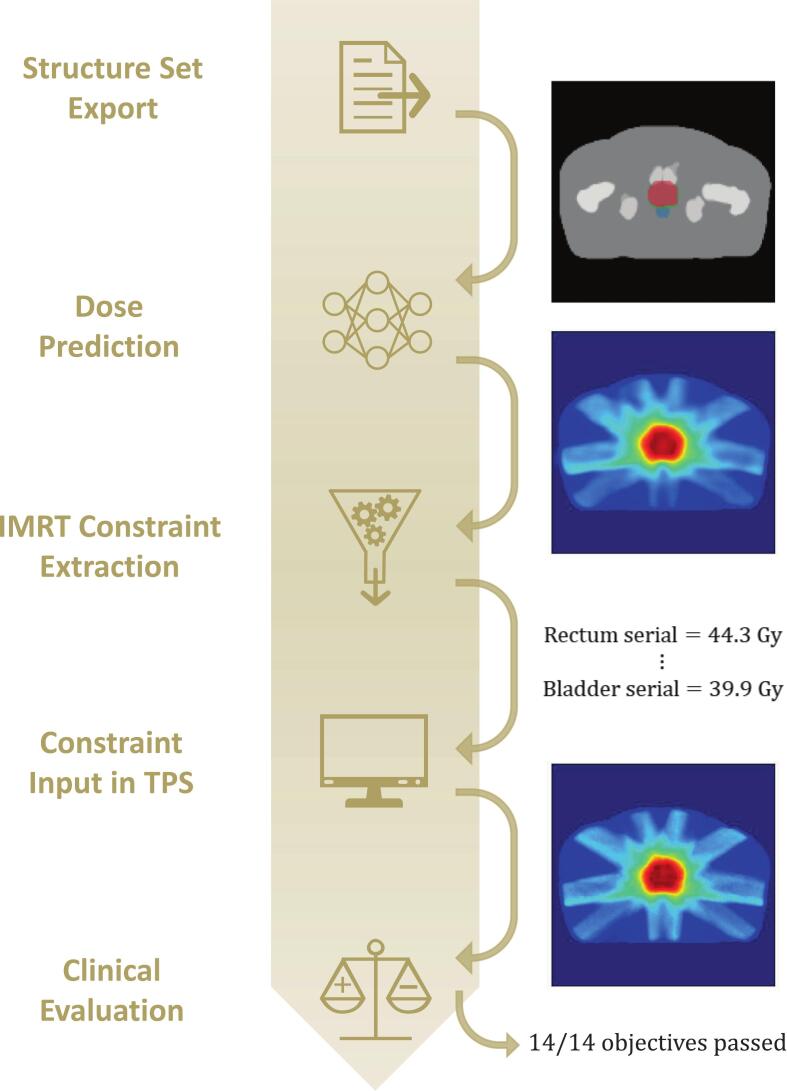
AutoAdapt pipeline.

Using this dose prediction model, a pipeline similar to Liu et al. was implemented, which can be used without access to the clinical TPS, as illustrated in Fig. 1 [28]. It uses the exported structure set from the daily MRI to calculate the occupancy maps that the TPS uses for plan optimization. These occupancy maps were used to create a population-based synthetic bulk density CT [31], which was then used to predict the dose distribution using the dose prediction model described in section 2.1.

The outputs of the prediction were used to calculate the serial and parallel plan constraints based on Alber et al. [32], included in a planning template used afterwards as input for the commercial TPS (cf. Supplementary Table S1). In the following, the different constraints are defined by OAR, constraint type (S for serial or P for parallel), and power law exponent used by the TPS as Rectum S12, S4, and P4, as well as Bladder S8 (cf. Supplementary material B). As the inference data was exported straight from the clinical setting, the pipeline was run via the central processing unit (Intel i7–12,700) on a clinical workstation.

### 2.3 Testing

The pipeline was tested on data from 10 patients with prostate cancer who were previously treated at the MR-Linac, consisting of a reference MRI scan and the first Adapt-to-Shape (ATS) fraction. All contours were retrospectively reviewed and adapted by a board-certified radiation oncologist. For a realistic comparison to the clinical adaptation workflow, including timing, two medical physicists with more than 3 years of experience in MRgRT treatment planning created reference plans for five patients each, using the AutoAdapt plan template. To account for the time between reference planning and the first fraction, including eventual staff rotation, the planners each adapted the plan where they were not responsible for the reference plan. Both planners were given the instructions to plan as they would do in clinical routine, with the goal of meeting all clinical objectives under simulated clinical time constraints of the adaptive workflow. All treatment plans were created using the TPS MONACO (Elekta, Stockholm, Sweden), Version 6.2.2.

### 2.4 Analysis

The need for any adaptation was measured by comparing the clinical criteria against the plan optimized based on the reference plan constraints. Deviations below 0.1 Gy were deemed as marginal but were still assessed by a board-certified radiation oncologist.

The manual-adapted and AutoAdapt plans were additionally compared based on total execution time and plan complexity. All DVH characteristics were evaluated based on median and range with respect to the clinical objectives (cf. Table 1), while testing for significance based on a two-sided Wilcoxon signed-rank test (α = 0.05). The plan constraint differences to the reference plan were compared between the manual and AutoAdapt methods to spot optimization consistency. Correlation analysis was performed using the Spearman correlation. To track the time consumption, the whole planning workflow for both methods was timed, including TPS optimization times to account for potentially longer optimization times due to higher plan complexity. For AutoAdapt, the time was additionally separated into Export, Preprocessing, Inference, Manual Input, and Calculation. Plan complexity was evaluated using the Plan Aperture Modulation (PAM) index, a metric introduced by Hernandez et al. [33] that measures the ratio of the planning target volume (PTV) that is blocked by the multi-leaf collimator in each segment.

**Table 1:** Performance of the AutoAdapt pipeline compared to manual planning and reference based on the clinical criteria. The *p*-values for the Wilcoxon signed rank test between AutoAdapt and manual planning were included in the last column. Clinical criteria with a significant difference of α < 0.05 were marked in bold.

Criterion	Objective	Median (range)			
		Reference	AutoAdapt	Manual	p=
**PTV60 D**_**2%**_	**< 64.2 Gy**	62.1 (61.8–62.5) Gy	62.2 (61.9–62.9) Gy	62.2 (61.9–62.7) Gy	0.178
**PTV60 D**_**50%**_	**> 60.0 Gy**	60.1 (59.8–60.3) Gy	60.1 (59.9–60.4) Gy	60.1 (60.1–60.3) Gy	0.477
**PTV60 D**_**98%**_	**> 57.0 Gy**	57.6 (54.8–58.3) Gy	57.8 (57.4–58.0) Gy	57.7 (57.4–58.1) Gy	0.879
**PTV57.6 D**_**50%**_	**> 57.6 Gy**	59.6 (59.3–59.9) Gy	59.6 (59.5–60.0) Gy	59.7 (59.6–59.9) Gy	0.094
**PTV57.6 D**_**98%**_	**> 54.7 Gy**	55.3 (53.4–55.7) Gy	55.1 (54.7–55.6) Gy	55.3 (54.8–55.5) Gy	0.340
**Rectum V**_**20Gy**_	**< 85%**	**43.4 (31.8–47.7) %**	**50.9 (36.6–73.6) %**	**44.0 (22.4–48.7) %**	**0.020**
**Rectum V**_**30Gy**_	**< 57%**	28.8 (19.9–35.9) %	28.9 (19.1–46.5) %	29.2 (15.3–35.5) %	0.232
**Rectum V**_**40Gy**_	**< 38%**	18.2 (12.9–25.7) %	17.9 (11.6–29.6) %	19.1 (11.0–24.9) %	0.984
**Rectum V**_**50Gy**_	**< 22%**	10.2 (7.3–15.4) %	9.5 (6.3–15.3) %	9.8 (6.7–14.3) %	0.246
**Rectum D**_**0.035cm**^**3**^_	**< 60 Gy**	**59.6 (58.9–60.1) Gy**	**59.1 (57.6–59.9) Gy**	**59.7 (59.0–60.0) Gy**	**0.010**
**Rectum circumference**	**< 30 Gy**	14.2 (6.1–27.0) Gy	17.9 (11.7–26.2) Gy	14.7 (6.7–28.8) Gy	0.131
**Bladder V**_**48Gy**_	**< 50%**	9.7 (3.5–16.6) %	11.2 (4.4–15.4) %	10.9 (4.1–15.1) %	0.557
**Bladder V**_**56Gy**_	**< 18%**	3.7 (1.5–6.2) %	4.4 (2.0–7.1) %	5.2 (2.0–6.0) %	0.977
**Bladder V**_**60Gy**_	**< 2 cm**^**3**^	0.4 (0–2.1) cm^3^	0.4 (0.1–2.1) cm^3^	0.5 (0.2–1.6) cm^3^	0.447

## 3 Results

Non-adapted plans met all clinical criteria in 4 patients, with 2 patients showing severe underdosages of more than 1 Gy, which were clinically unacceptable. While AutoAdapt achieved all clinical criteria in seven of the patients, all treatment plans were rated clinically acceptable by a board-certified radiation oncologist without further adaptations (Table 1). The PTV60 objective for D_50%_ was not fulfilled in two patients, with only achieving 59.9 Gy, and bladder V_60Gy_ was breached with 2.1 instead of 2.0 cm^3^ in one other patient, all being characterized as marginal deviations.

All manual plans met all clinical objectives with no quality difference between planers. Manual plans achieved a significantly lower rectum V_20Gy_ compared to AutoAdapt (*p* = 0.02), whereas AutoAdapt significantly reduced rectum D_0.035cm^3^_ (*p* = 0.01). There was no significant difference observed for any of the bladder or PTV criteria (Table 1).

**Fig. 2:**
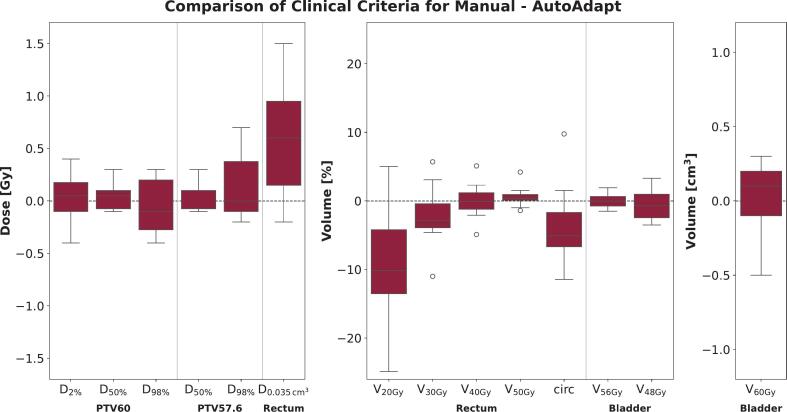
Box plots of differences in clinical criteria between manual planning and AutoAdapt pipeline using quartile and median values. A positive value equals a higher value in manual planning.

**Fig. 3:**
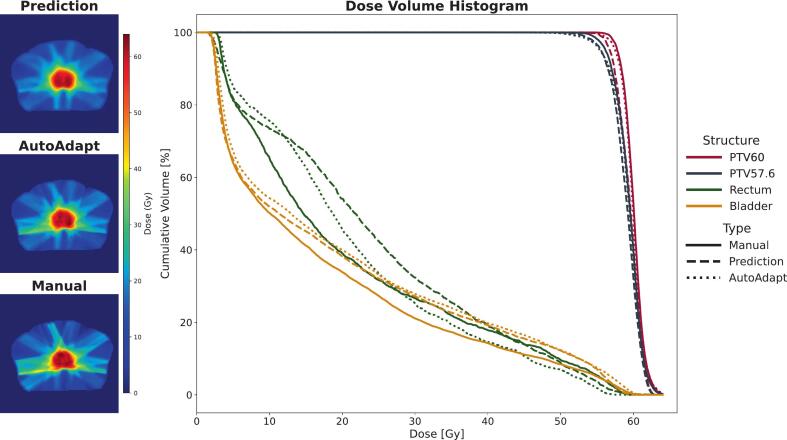
Patient example for dose distribution of prediction, AutoAdapt and manual planning and the corresponding dose volume histogram.

In the patient-specific analysis comparing both methods all clinical PTV criteria stayed within a difference of 0.5 Gy (Fig. 2). Rectum V_20Gy_ showed lower values when manually planned, up to 25%, with a median difference of over 10%. In comparison, AutoAdapt showed a lower V_50Gy_ of up to 4.2% with an absolute median difference of 0.4%. The rectum D_0.035cm^3^_ resulted in a median absolute dose difference of 0.6 Gy. The results from AutoAdapt were similar to the initial prediction results for bladder and target volumes; for some patients AutoAdapt achieved lower rectum low dose values (Fig. 3).

**Fig. 4:**
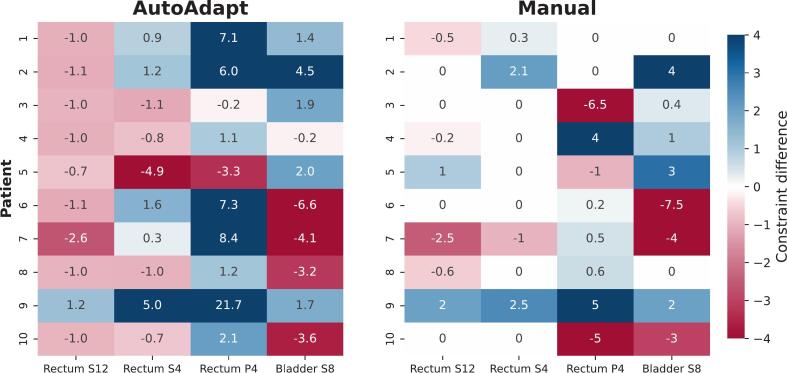
Changes in plan constraints (Rectum S12, Rectum S4, Rectum P4, and Bladder S8) compared to the reference plan for manual planning and AutoAdapt on a patient's individual basis. A strengthening of the constraint compared to the reference plan is indicated in red, while a loosening is indicated in blue. (For interpretation of the references to colour in this figure legend, the reader is referred to the web version of this article.)

When assessing the plan constraint difference compared to the reference plan created by manual planners to characterize feature extraction of the model, a higher likelihood for adaptation could be spotted for AutoAdapt (Fig. 4). The Spearman correlation comparing changes with respect to the reference plan between AutoAdapt and manual planning showed a very strong correlation of 0.93 for the serial bladder constraint (*p* < 0.001) and a strong correlation of 0.66 for the high serial rectum constraint (*p* = 0.036). Both rectum constraints managing low rectum dose (Parallel 4 and Serial 4) showed only moderate correlation of 0.5 and 0.54 (*p* = 0.14, 0.11). The two patients with the greatest loosening of the plan constraints both severely underdosed the PTV when using the reference constraints.

**Fig. 5:**
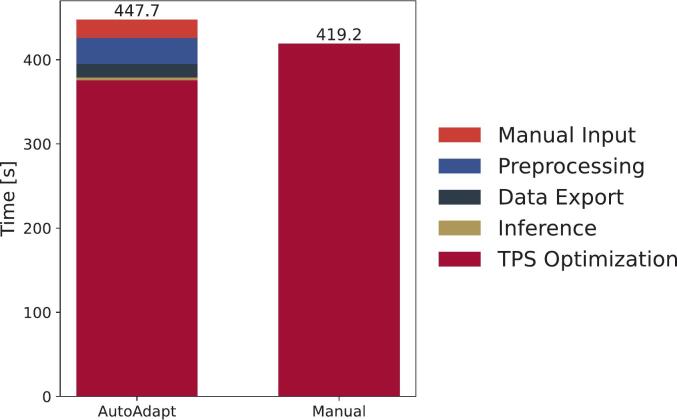
Comparison between the planning time for manual planning and AutoAdapt pipeline. The AutoAdapt workflow time is subdivided into data export, preprocessing, inference, and manual input by the user.

When comparing the time expense for both methods, as illustrated in Fig. 5, AutoAdapt used a higher mean time. The actual TPS Optimization time is 44 s longer for manual planning, while the extra steps in AutoAdapt take an additional 72 s. Especially the preprocessing and manual input of the constraints into the TPS lead to slightly increased processing times for AutoAdapt. The actual mean inference time was only 3.2 s. The plan complexity was significantly lower in AutoAdapt with a mean PAM of 0.276 compared to 0.322 in manual plans (*p* = 0.007), which resulted in a median monitor unit decrease of 6.4% (*p* = 0.006).

## 4 Discussion

The AutoAdapt pipeline using a physics-assisted dose prediction model proved to be able to efficiently adapt treatment plans for MRgRT with minimal user interaction and time requirements. When comparing the performance of manual and AutoAdapt plans to the plans based on the reference constraints, a need for adaptation could be illustrated, especially in cases where the constraints needed to be loosened. Although three cases negligibly breached some of the objectives in AutoAdapt, all plans were still clinically acceptable.

A reason for the different performances of manual and AutoAdapt for different objectives might be the automatically generated PSO treatment plan dataset used for model training, which seems to emphasize the rectum V20Gy less, to focus on higher dose coverage and improved rectum D_0.035cm^3^_. This is also consistent with the constant constraint shift in the high-dose rectum constraint.

While the dose prediction model seemed to value different criteria compared to the manual planners, when it came to large changes where an adaptation was necessary, they agreed on what to change, as indicated by the high Spearman correlation in the bladder constraint. This could be especially valuable as it indicates the capability of AutoAdapt to detect, where an adaptation is even necessary.

Unfortunately, the manual planners did not try to adapt all four constraints for all patients and instead focused only on the ones they deemed important, rendering a complete comparison difficult and risking missing out on small adjustments as done by AutoAdapt.

An additional cause for the lower rectum V_20Gy_ could be the rather small dataset, as prior studies already showed that increasing the dataset can lead to an increase in quality in low-dose areas. Van Genderingen et al. [34] state that the dataset needs at least 250 datapoints to show a passable low-dose contrast. Although the dataset has a lot of data points, they are from a small patient population, leading to less information gain than data points from different patients.

One of the biggest advantages of AutoAdapt was the reduction of planner time. Although AutoAdapt took slightly longer than manual optimization, the planner only needed to input the constraints at the beginning, taking less than 20 s instead of constantly monitoring and adapting the changes over the whole optimization process in manual planning. This becomes even more important in regard to the implementation of distributed planning, where the planner cannot interact in the middle of the optimization due to the optimization taking place on a separate computing server. AutoAdapt could directly give the optimal start constraints, while the manual planner would need multiple optimization cycles to find an adequate solution.

The added bonus of lower optimization times and lower Monitor Unit usage in AutoAdapt resulted most likely from the lower emphasis on sparing the rectum wall and the fact that the constraints were static throughout the whole optimization, making it easier for the program to converge on a solution.

It was demonstrated that a population-based dose prediction method could be used to run the AutoAdapt pipeline in a clinical setting by comparison with the results of Liu et al. [28]. While not using patient-specific information from earlier fractions, the method has been shown to be more flexible and could thus potentially also be used to create reference plans, saving additional time.

There was, however, no improvement in planning time, as the planning process was finished after one optimization cycle in all patients, with the additional time needed for export and preprocessing taking additional time and thus currently compensating for optimization time gains.

Naccarato et al. [25] presented an alternative integrated solution that accesses the optimization process, leading to fast and high-quality plans. By gaining access to the optimizer results, dose predictions could be used in a similar manner to guide the optimization on a voxel basis. Similarly, Khalifa et al. [26] demonstrated the feasibility of such an approach when employing an external dose-mimicking algorithm (RayStation, RaySearch, Stockholm, Sweden). Further refinement is, however, necessary as the dose calculation used in this study did not account for the effects of magnetic fields.

To further increase time gain, the plan segmentation creating the actual treatment plan would need to be accelerated as well. While this is done in MRgRT via GPU-based Monte Carlo simulation, an AI-based plan segmentation tool may lead to significant time gains. Heilemann et al. [35] showed that a sub-minute optimization of a treatment plan is possible. While those results were achieved for a volumetric modulated arc therapy plan on a conventional linear accelerator, a transfer of the method to other IMRT techniques seems possible. For MRgRT, Buchanan et al. [36] already used fluence distributions to create applicable treatment plans. By linking dose distribution to fluence, a complete AI-based treatment optimization for MRgRT could be achieved.

AutoAdapt is, similar to other AI methods, heavily dependent on the training set and thus offers limited potential to individualize dose planning. After creating a dose prediction model, any adaptation to the plan template in the form of beam setup or fractionation concept would need a retraining and commissioning of the model. While this is easy for already established fractionation schemes, new additions would require an extensive data collection phase before they could be implemented into clinical routine. If applied to out-of-distribution cases, AutoAdapt running completely autonomously could risk pipeline failure, costing valuable time in the clinical setting. A semi-hands-off planning assisted by the pipeline could be a valuable solution to reduce planner load while retaining high quality and safety standards.

In conclusion, a pipeline was developed that delivers high-quality plans in a comparable time to the clinical standard, while reducing the burden on the treatment planner. By including a physical loss component and using fixed beam directions, even a small patient cohort was sufficient to train a usable population-based dose prediction model. As the planer can still interact with the TPS throughout the optimization process and adjust if necessary, making it a versatile option for hands-off or semi-hands-off adaptation.

## Declaration of generative AI and AI-assisted technologies in the manuscript preparation process

During the preparation of this work the authors used Grammerly and DeepL in order to improve the readability of the manuscript. After using these tools, the authors reviewed and edited the content as needed and takes full responsibility for the content of the published article.

## CRediT authorship contribution statement

**Benjamin Tengler:** Writing – original draft, Visualization, Validation, Software, Investigation, Formal analysis, Data curation. **Moritz Schneider:** Writing – review & editing, Validation, Resources, Data curation. **Marcel Nachbar:** Writing – review & editing, Validation, Resources, Data curation, Conceptualization. **Simon Boeke:** Writing – review & editing, Validation, Resources, Data curation. **Cihan Gani:** Writing – review & editing, Validation, Resources, Data curation. **Maximilian Niyazi:** Writing – review & editing, Resources, Funding acquisition. **Paul Fischer:** Writing – review & editing, Software, Methodology. **Christian F. Baumgartner:** Writing – review & editing, Software, Methodology. **Daniela Thorwarth:** Writing – review & editing, Supervision, Funding acquisition, Conceptualization.

## Declaration of competing interest

The authors declare the following financial interests/personal relationships which may be considered as potential competing interests: Funding: DFG (ZI 736/2-1). COI: research collaborations Elekta AB, Philips, PTW Freiburg, Dr. Sennewald Medizintechnik, Kaiku Health, TheraPanacea, ITV, Brainlab. S. Boeke received funding by Merck (speaker fees, travel expenses), Elekta (speaker fees, travel expenses) and Regeneron (speaker fees).
