# Supplementary material for: Development of an artificial intelligence driven dose prediction pipeline for online adaptive magnetic resonance-guided radiotherapy
Source: Phys Imaging Radiat Oncol. 2026 Jul 23;40:101048. doi: 10.1016/j.phro.2026.101048 (PMC13449337; doi:10.1016/j.phro.2026.101048)

Table S1: Plan constraint template used for all treatment plans. Constraints acquired via constraint calculation are marked in orange.

Structure	Type	Reference dose	Exponent	Shrink [mm]	Constraint
PTV60	Target EUD				60
	Target Penalty				57
	Quadratic Overdose	60			0.8
PTV57.6	Target EUD			0	57.6
	Target Penalty				54.7
	Quadratic Overdose	60		0	0.15
	Quadratic Overdose	57.6		3	0.6
Rectum	Serial		12		Dose prediction
	Serial		4	0	Dose prediction
	Parallel	30	4	0	Dose prediction
	Quadratic Overdose	59			0.05
Bladder	Serial		8		Dose prediction
	Quadratic Overdose	59.8			0.04
FemoralHead_R	Quadratic Overdose	50			0.2
FemoralHead_L	Quadratic Overdose	50			0.2
Urethra	Quadratic Overdose	60			0.2
PenileBulb	Parallel	40	3	3	50
patient	Quadratic Overdose	56		0	0.05
	conformal				1

Supplementary material A:

Swin UNEt Transformer (Swin UNETR)

The Swin UNet Transformer is an architecture initially created for the 2021 Brats Challenge by Nvidia, which achieved seventh place. It combines the capabilities of UNets to morph 2D and 3D images with Transformer capabilities in modeling long-range information [1]. Even with small kernel sizes, information can be distributed longer and faster, giving the possibility to downscale models.

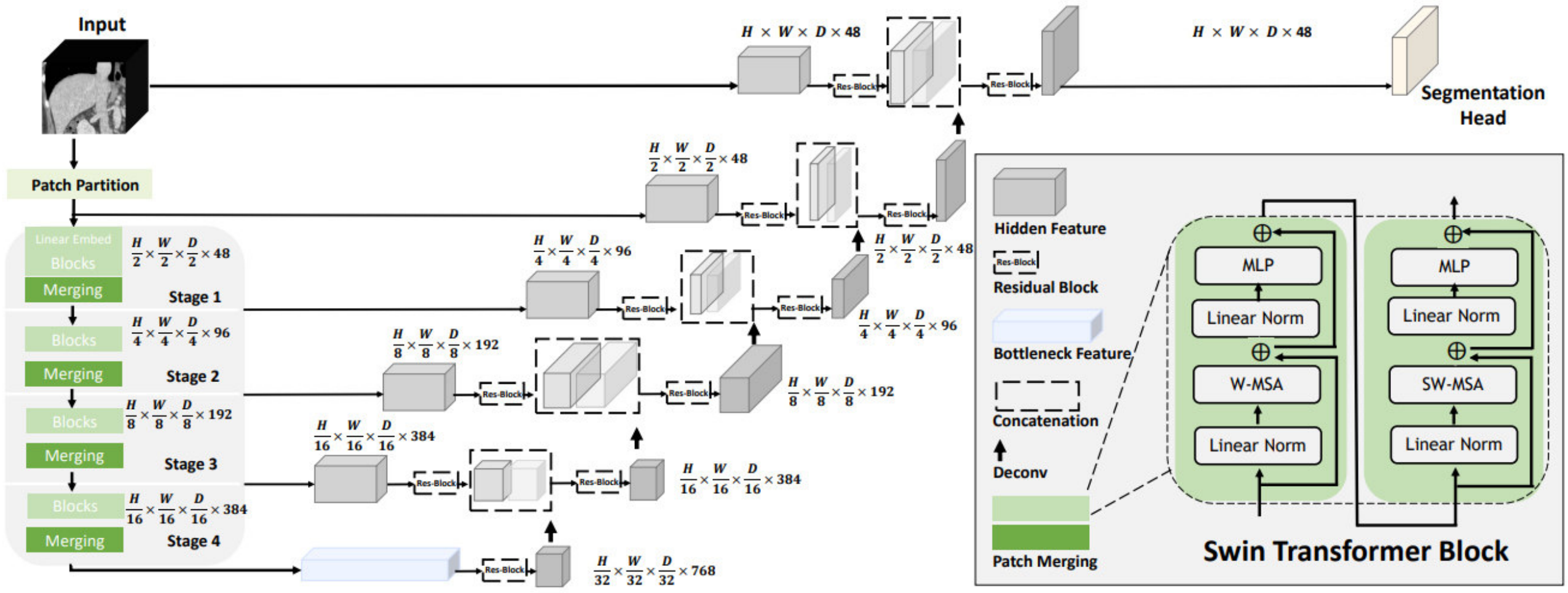

Figure S1: Original Swin UNETR Architecture by Hatamizadeh et al. [1].

The model consists of a Sliding Window (Swin) Transformer as an encoder, which uses multiple transformer blocks to subsample the data while transposing the sample grid to improve long-range information transfer [2]. The decoder follows a standard UNet shape.

Modifications to the Swin UNETR:

While the standard architecture consists of 4 layers with an input mask of 128 x 128 x 128, we needed to adapt the model to accept an input of 144 x 144 x 96. We decided to decrease the model depth to three layers to limit the risk of overfitting due to our small dataset.

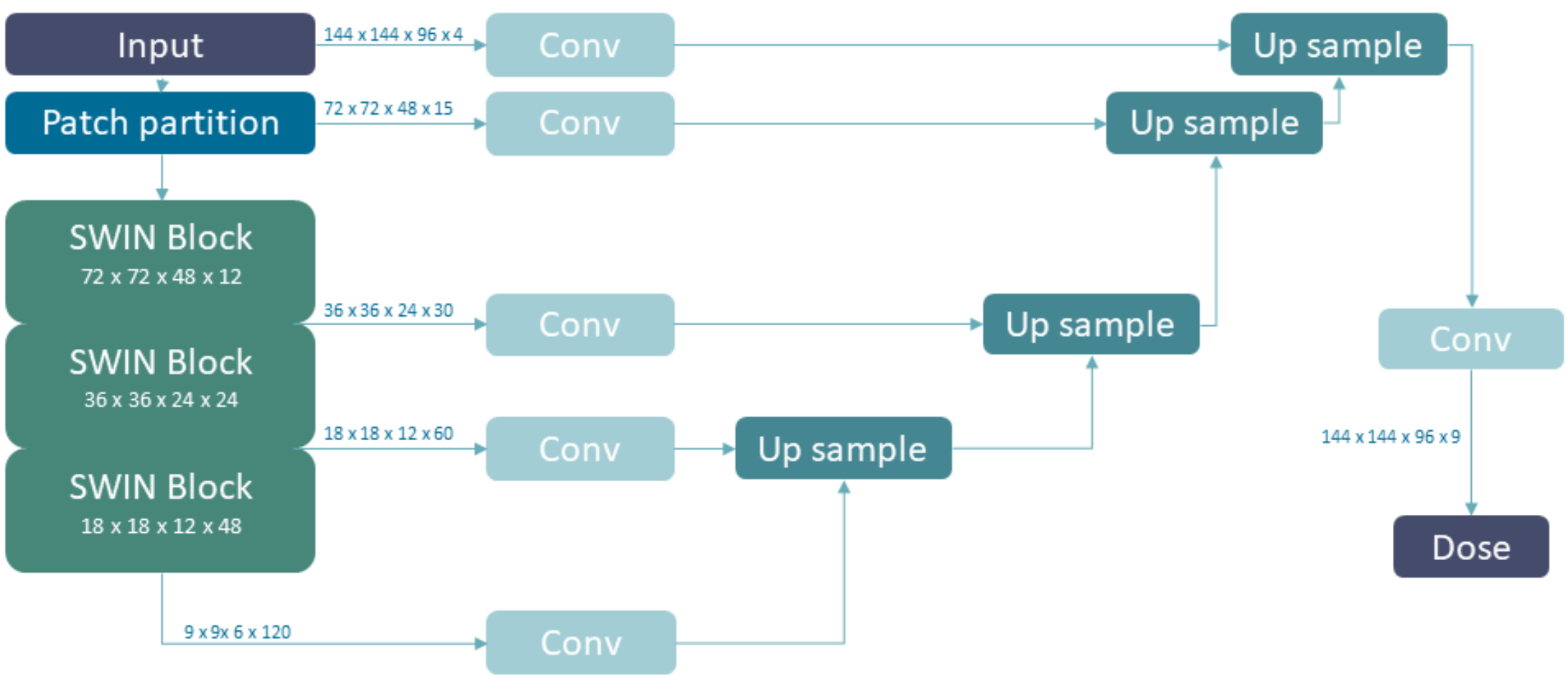

Figure S2: Adapted Swin UNETR for this study

Supplementary material B:

Plan constraint calculation

The constraints for the plan adaptation were calculated using Alber et al. [3]:

Parallel:

$$V_{parallel} = \sum V(D) \frac{1}{1 + \frac{D_0}{D}^k}$$

Serial:

$$D_{serial} = \sqrt[k]{\sum V(D) * D^k}$$

with k being the exponent and D_0 defining the reference dose.

References

[1] Hatamizadeh A, Nath V, Tang Y, Yang D, Roth HR, Xu D. Swin UNETR: Swin Transformers for Semantic Segmentation of Brain Tumors in MRI Images. Lect Notes Comput Sci. Cham: Springer International Publishing; 2022. p. 272–84 https://doi.org/10.1007/978-3-031-08999-2_22.

[2] Liu Z, Lin Y, Cao Y, Hu H, Wei Y, Zhang Z, et al. Swin transformer: Hierarchical vision transformer using shifted windows. Proc IEEE/CVF Int Conf Comput Vis2021. p. 10012–22 https://doi.org/10.1109/ICCV48922.2021.00986.

[3] Alber M. Normal tissue dose-effect models in biological dose optimisation. Z Med Phys. 2008;18:102–10 https://doi.org/10.1016/j.zemedi.2007.08.002.

Model Card — version 1.0 — Model AutoAdapt

Task: Dose prediction

---

0. Card Metadata

Creation date * : 2025/12/08

Versioning

- **Version number *** : 1.0
- **Version changes *** : Introduction

---

1. Model Basic Information

Name * : AutoAdapt

Creation date * : 2025/11/05

Versioning

- **Version number *** : 01.00.0000
- **Version changes *** : First Model

Model scope

- **Summary *** : Dose prediction pipeline to predict plan parameters for a prostate cancer patient treated with 20 times 3/2.67 Gy (60/57.6 Gy) using a plan template.
- **Anatomical site *** : Pelvis

Clearance

- **Type *** : only research use

Approved by

- **Name(s)**: NA
- **Institution(s) *** : NA
- **Contact email(s)**: —

Intended users: radiation technologists, medical physicists

Observed limitations * : Limited low dose accuracy, not implemented for implants

Potential limitations: Danger of out of distribution patients due to low patient cohort

Type of learning architecture * : Swin Unet

Developed by

- **Name**: Benjamin Tengler
- **Institution(s) *** : University of Tübingen
- **Contact email(s)**: benjamin.tengler@med.uni-tuebingen.de

Conflict of interest * : NA

Software licence * : Apache 2.0

Code source: MONAI

Citation details: Model adapted from SWIN Unet MONAI

URL info: https://github.com/Project-MONAI/MONAI/blob/dev/monai/networks/nets/swin_unetr.py

---

2. Technical specifications

2.1 Model overview

Model pipeline

- **Summary *** : DICOM Structuresets are preprocessed to input maps of ize 144 x 144 x 96 with a voxel size of 3 x 3 x 3 mm. The stucture sets are translated into a percentage vize occupancy map in a scale of 0 to 1. The Inputs are: PTV, Rectum, Bladder, Urethra map and bulk density assigned CT for bones, patients, Rectum and Bladder.
- **Model inputs *** : ['RTSTRUCT: PTV, Rectum, Bladder, Urethra, External, Bone_Pelvic_L, Bone_Pelvic_R, Femur_L, Femur_R, Sacrum']
- **Additional information**: —
- **Model outputs *** : ['Plan Parameters']
- **Additional information**: —
- **Pre-processing *** : RTStruct to Occupancy maps with voxel size 3 x 3 x 3 mm.
- **Post-processing *** : Calculation of 4 parameters rounded to 2 decimals

2.2 Learning architecture(s)

Learning architecture 1

Field	Value
**Total number of trainable parameters ***	3782326
**Number of inputs**	5
**Input content**	—
**Input format**	float
**Input size**	[144,144,96]

Field	Value
**Number of outputs**	9
**Output content**	—
**Output format**	float
**Output size**	[9,144,144,96]
**Loss function ***	10(*0.02*RMSE_total+RMSE)<br>+0.02*(DVH_Loss+Parameter_Loss)+5*Gradient_Loss
**Batch size**	2
**Regularisation**	Dropout
**Uncertainty quantification techniques ***	NaN
**Explainability techniques ***	NaN

2.3 Hardware & software

- **Libraries and dependencies:** MONAI
- **Hardware (recommended):** GPU 10 GB but only tested on CPU
- **Inference time (s) for recommended hardware:** 80.0

---

3. Training Data Methodology and Information

Fine tuned form

- **Model name *** : NA
- **URL/DOI to model card *** : NA
- **Tuning technique *** : NA

Training Dataset

General information

- **Total size *** : [216]
- **Number of patients *** : 18
- **Source *** : Private dataset by the University Clinic of Tübingen
- **Acquisition period *** : November 2019 - November 2021
- **Inclusion / exclusion criteria *** : only patients treated 20 times with 3/2.67 Gy. No implants
- **Type of data augmentation**: NA
- **Strategy for data augmentation:** NA

Technical specifications

Plan Parameters (model_outputs)

Field	Value
**Image resolution ***	[3,3,3]
**Patient positioning ***	[Supine]
**Scan(s) manufacturer and model ***	NA
**Scan acquisition parameters ***	NA
**Scan reconstruction parameters ***	NA
**FOV ***	[432,432,288]

RTSTRUCT: PTV, Rectum, Bladder, Urethra, External, Bone_Pelvic_L, Bone_Pelvic_R, Femur_L, Femur_R, Sacrum (model_inputs)

Field	Value
**Image resolution ***	[3,3,3]
**Patient positioning ***	[Supine]
**Scan(s) manufacturer and model ***	NA
**Scan acquisition parameters ***	NA
**Scan reconstruction parameters ***	NA
**FOV ***	[432,432,288]

- **Treatment modality *** : ['External beam radiation therapy - Photons - IMRT']
- **Beam configuration and energy *** : 9- beam IMRT 7 MV at 25°, 60°, 100°, 135°, 180°, 225°, 260°, 300°, 340°.
- **Dose engine *** : ['Monte Carlo']
- **Target volumes and prescription *** : [[PTV_60,PTV_57.6]]
- **Number of fractions *** : [20]
- **Reference standard *** : clinical contours by board-certified oncologist
- **Reference standard QA *** : delinations created/checked by board-certified oncologist

Patient demographics and clinical characteristics

- **ICD10/11**: 2C82.0
- **Age *** : NA
- **Sex *** : male

Validation strategy * : Basic Validation

Validation data partition * : [19%]

Epochs: [1164] **Optimiser**: AdamW **Learning rate**: 0.001

Model choice criteria * : early stopping after 500 epochs with no improvment in the validation set

Inference method * : single fold

---

4. Evaluation Data Methodology, Results and Commissioning

1 First Evaluation

Evaluation date * : 2025/11/11

Evaluated by

- **Name(s) *** : Benjamin Tengler
- **Institution(s) *** : University of Tübingen
- **Contact email(s) *** : benjamin.tengler@med.uni-tuebingen.de
- **Same as 'Approved by':** No

Evaluation frame * : retrospective

Evaluation dataset

General information

- **Total size *** : [50]
- **Number of patients *** : 8
- **Source *** : Clinical patients from the University Clinic Tübingen
- **Acquisition period *** : May 2021-November 2021
- **Inclusion / Exclusion criteria *** : Patients treated with 7 times 6.1 Gy that were recontoured for 20 times 3 Gy.
- **URL info**: —

Technical specifications

Plan Parameters (model_outputs)

Field	Value
**Image resolution ***	[3,3,3]
**Patient positioning ***	[Supine]
**Scan(s) manufacturer and model ***	NA
**Scan acquisition parameters ***	NA
**Scan reconstruction parameters ***	NA
**FOV ***	[432,432,288]

RTSTRUCT: PTV, Rectum, Bladder, Urethra, External, Bone_Pelvic_L, Bone_Pelvic_R, Femur_L, Femur_R, Sacrum (model_inputs)

Field	Value
**Image resolution ***	[3,3,3]
**Patient positioning ***	[Supine]
**Scan(s) manufacturer and model ***	NA
**Scan acquisition parameters ***	NA
**Scan reconstruction parameters ***	NA
**FOV ***	[432,432,288]

- **Dose engine *** : ['Monte Carlo']
- **Target volumes and prescription *** : [[PTV_60,PTV_57.6]]
- **Number of fractions *** : [20]
- **Reference standard *** : delieated under supervision of the board certified oncologist
- **Reference standard QA *** : checked by board certified oncologist

Patient demographics and clinical characteristics

- **ICD10/11**: 2C82.0
- **Age *** : NA
- **Sex *** : male

Quantitative evaluation

Dose Metrics

D2%

Field	Value
**Type ***	D2%
**Metric Specifications ***	<64.2Gy
**On Volume ***	PTV_60
**Dose Grid Resolution ***	[3,3,3]
**Sample Data**	—
**Mean Data ***	[62.2,61.9,62.9,0.0]
**Figure DM DP Appendix Label**	Fig 2

D50%

Field	Value
**Type ***	D50%
**Metric Specifications ***	>60.0Gy
**On Volume ***	PTV_60
**Dose Grid Resolution ***	[3,3,3]
**Sample Data**	—
**Mean Data ***	[60.1,59.9,60.4,0.0]
**Figure DM DP Appendix Label**	Fig 2

D98%

Field	Value
**Type ***	D98%
**Metric Specifications ***	>57.0Gy
**On Volume ***	PTV_60
**Dose Grid Resolution ***	[3,3,3]
**Sample Data**	—
**Mean Data ***	[57.8,57.4,58.0,0.0]
**Figure DM DP Appendix Label**	Fig 2

D50%

Field	Value
**Type ***	D50%
**Metric Specifications ***	>57.6Gy
**On Volume ***	PTV_57.6
**Dose Grid Resolution ***	[3,3,3]
**Sample Data**	—
**Mean Data ***	[59.6,59.5,60.0,0.0]
**Figure DM DP Appendix Label**	Fig 2

D98%

Field	Value
**Type ***	D98%

Field	Value
**Metric Specifications ***	>54.7Gy
**On Volume ***	PTV_57.6
**Dose Grid Resolution ***	[3,3,3]
**Sample Data**	—
**Mean Data ***	[55.1,54.7,55.6,0.0]
**Figure DM DP Appendix Label**	Fig 2

V20Gy

Field	Value
**Type ***	V20Gy
**Metric Specifications ***	<85%
**On Volume ***	Rectum
**Dose Grid Resolution ***	[3,3,3]
**Sample Data**	—
**Mean Data ***	[50.9,36.6,73.6,0.0]
**Figure DM DP Appendix Label**	Fig 2

V30Gy

Field	Value
**Type ***	V30Gy
**Metric Specifications ***	<57%
**On Volume ***	Rectum
**Dose Grid Resolution ***	[3,3,3]
**Sample Data**	—
**Mean Data ***	[28.9,19.1,46.5,0.0]
**Figure DM DP Appendix Label**	Fig 2

V40Gy

Field	Value
**Type ***	V40Gy
**Metric Specifications ***	<38%

Field	Value
**On Volume ***	Rectum
**Dose Grid Resolution ***	[3,3,3]
**Sample Data**	—
**Mean Data ***	[17.9,11.6,29.6,0.0]
**Figure DM DP Appendix Label**	Fig 2

V50Gy

Field	Value
**Type ***	V50Gy
**Metric Specifications ***	<22%
**On Volume ***	Rectum
**Dose Grid Resolution ***	[3,3,3]
**Sample Data**	—
**Mean Data ***	[9.5,6.3,15.3,0.0]
**Figure DM DP Appendix Label**	Fig 2

D0.035ccm

Field	Value
**Type ***	D0.035ccm
**Metric Specifications ***	<60Gy
**On Volume ***	Rectum
**Dose Grid Resolution ***	[3,3,3]
**Sample Data**	—
**Mean Data ***	[59.1,57.6,59.9,0.0]
**Figure DM DP Appendix Label**	Fig 2

Circumference

Field	Value
**Type ***	Circumference
**Metric Specifications ***	<30Gy
**On Volume ***	Rectum

Field	Value
**Dose Grid Resolution ***	[3,3,3]
**Sample Data**	—
**Mean Data ***	[17.9,11.7,26.2,0.0]
**Figure DM DP Appendix Label**	Fig 2

V48Gy

Field	Value
**Type ***	V48Gy
**Metric Specifications ***	<50%
**On Volume ***	Bladder
**Dose Grid Resolution ***	[3,3,3]
**Sample Data**	—
**Mean Data ***	[11.2,4.4,15.4,0.0]
**Figure DM DP Appendix Label**	Fig 2

V56Gy

Field	Value
**Type ***	V56Gy
**Metric Specifications ***	<18%
**On Volume ***	Rectum
**Dose Grid Resolution ***	[3,3,3]
**Sample Data**	—
**Mean Data ***	[4.4,2.0,7.1,0.0]
**Figure DM DP Appendix Label**	Fig 2

V60Gy

Field	Value
**Type ***	V60Gy
**Metric Specifications ***	<2ccm
**On Volume ***	Rectum
**Dose Grid Resolution ***	[3,3,3]

Field	Value
**Sample Data**	—
**Mean Data ***	[0.4,0.1,2.1,0.0]
**Figure DM DP Appendix Label**	Fig 2

Qualitative evaluation

Evaluators information * : one physicist with 6 years of treatment planning experience

Likert scoring

- **Method *** : NA
- **Results *** : NA

Turing test

- **Method *** : NA
- **Results *** : NA

Time saving

- **Method *** : NA
- **Results *** : NA

Other

- **Method *** : —
- **Results *** : —

Explainability * : NA

Citation details: —

---

5. Other considerations

- **Risk Analysis:** the optimization is done via the clinical TPS and the finished plan needs to be reviewed by the oncologist
- **Post-Market Surveillance / Live Monitoring:** Recommended to have a medical physicist for fast modifications if AI fails.

Appendix

Fig 1: training_curve.png

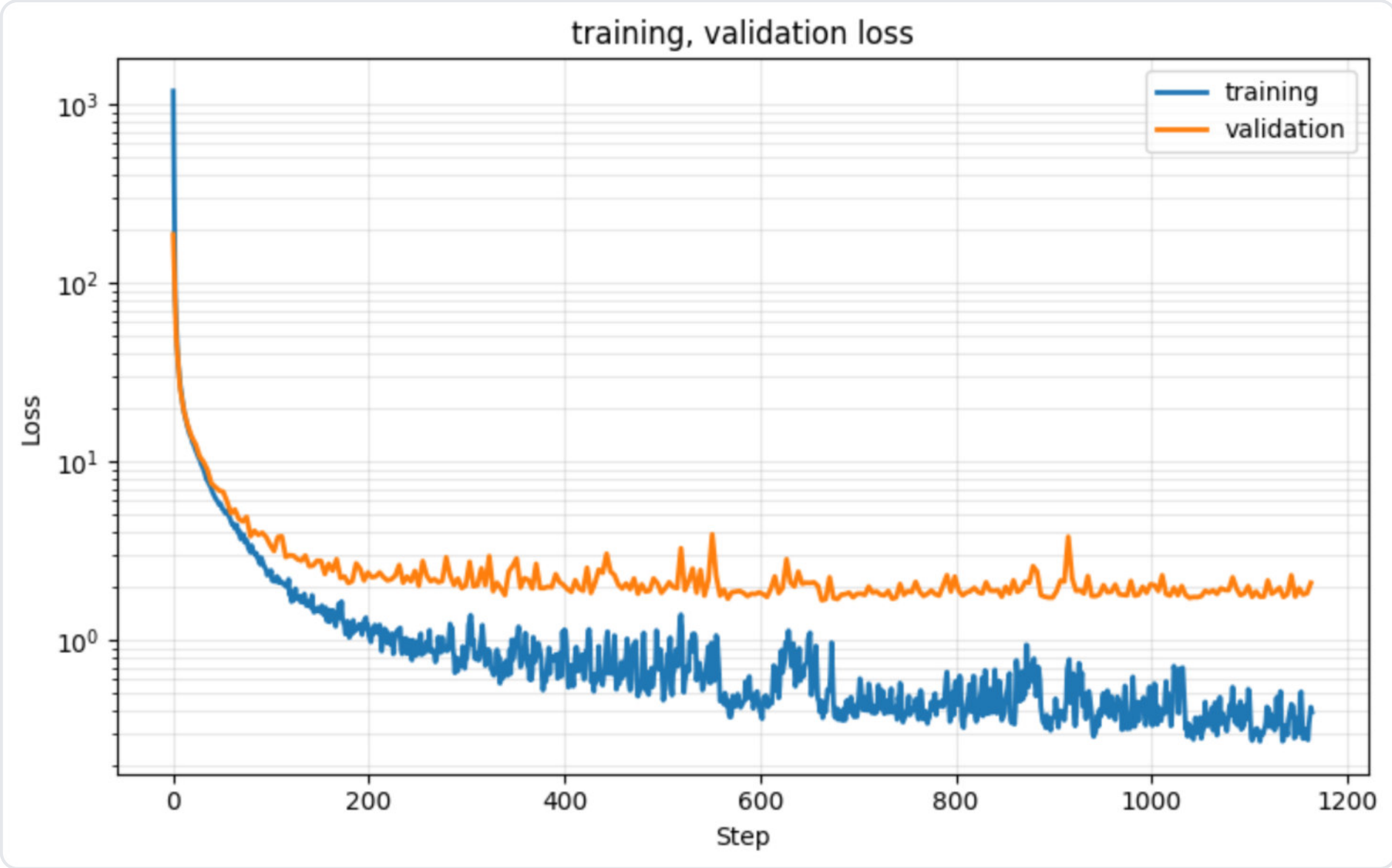

Fig 2: table_DVH.png

Criterion	Objective	Median (range)		
		AutoAdapt	Manual	p=
PTV60 $D_{2\%}$	< 64.2 Gy	62.2 (61.9-62.9) Gy	62.2 (61.9-62.7) Gy	0.178
PTV60 $D_{50\%}$	> 60.0 Gy	60.1 (59.9-60.4) Gy	60.1 (60.1-60.3) Gy	0.477
PTV60 $D_{98\%}$	> 57.0 Gy	57.8 (57.4-58.0) Gy	57.7 (57.4-58.1) Gy	0.879
PTV57.6 $D_{50\%}$	> 57.6 Gy	59.6 (59.5-60.0) Gy	59.7 (59.6–59.9) Gy	0.094
PTV57.6 $D_{98\%}$	> 54.7 Gy	55.1 (54.7–55.6) Gy	55.3 (54.8–55.5) Gy	0.340
Rectum V_{20Gy}	< 85%	**50.9 (36.6–73.6) %**	**44.0 (22.4–48.7) %**	**0.020**
Rectum V_{30Gy}	< 57%	28.9 (19.1–46.5) %	29.2 (15.3–35.5) %	0.232
Rectum V_{40Gy}	< 38%	17.9 (11.6–29.6) %	19.1 (11.0–24.9) %	0.984
Rectum V_{50Gy}	< 22%	9.5 (6.3–15.3) %	9.8 (6.7–14.3) %	0.246
Rectum $D_{0.035\,ccm}$	< 60 Gy	**59.1 (57.6–59.9) Gy**	**59.7 (59.0–60.0) Gy**	**0.010**
Rectum circumference	< 30 Gy	17.9 (11.7–26.2) Gy	14.7 (6.7–28.8) Gy	0.131
Bladder V_{48Gy}	< 50%	11.2 (4.4–15.4) %	10.9 (4.1–15.1) %	0.557
Bladder V_{56Gy}	< 18%	4.4 (2.0–7.1) %	5.2 (2.0–6.0) %	0.977
Bladder V_{60Gy}	< 2 ccm	0.4 (0.1–2.1) ccm	0.5 (0.2–1.6) ccm	0.447

Fig 3: Architecture_SWIN_Unetr.png

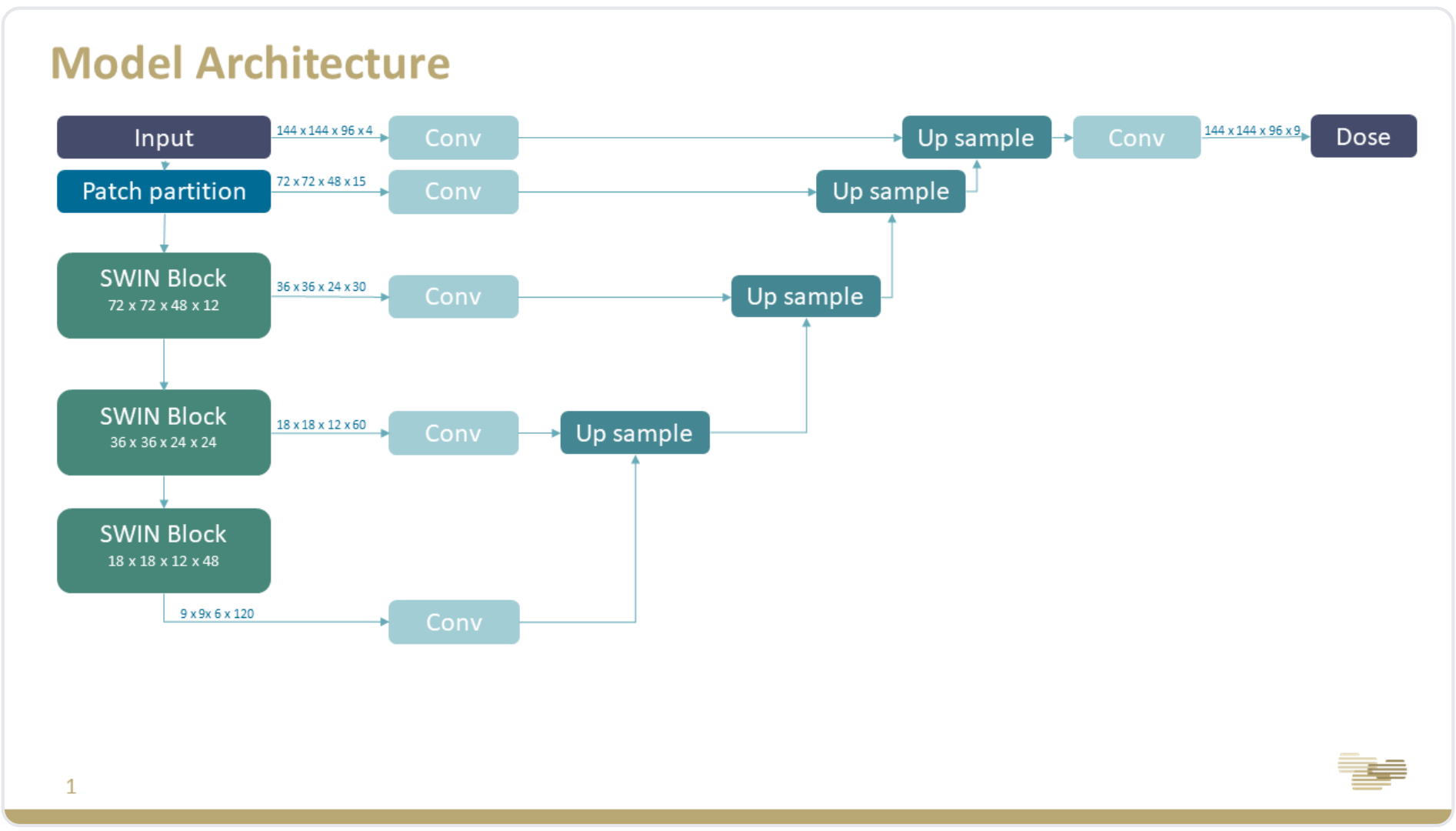

Supplement: Supplementary file 1 [file mmc1.pdf]
